# The Role of Glypican-3 in Regulating Wnt, YAP, and Hedgehog in Liver Cancer

**DOI:** 10.3389/fonc.2019.00708

**Published:** 2019-08-02

**Authors:** Aarti Kolluri, Mitchell Ho

**Affiliations:** ^1^Laboratory of Molecular Biology, Center for Cancer Research, National Cancer Institute, National Institutes of Health, Bethesda, MD, United States; ^2^Mayo Clinic Graduate School of Biomedical Sciences, Rochester, MN, United States

**Keywords:** Wnt signaling, heparan sulfate proteoglycan, antibody therapy, liver cancer, YAP signaling

## Abstract

Glypican-3 (GPC3) is a cell-surface glycoprotein consisting of heparan sulfate glycosaminoglycan chains and an inner protein core. It has important functions in cellular signaling including cell growth, embryogenesis, and differentiation. GPC3 has been linked to hepatocellular carcinoma and a few other cancers, however, the mechanistic role of GPC3 in cancer development remains elusive. Recent breakthroughs including the structural modeling of GPC3 and GPC3–Wnt complexes represent important steps toward deciphering the molecular mechanism of action for GPC3 and how it may regulate cancer signaling and tumor growth. A full understanding of the molecular basis of GPC3-mediated signaling requires elucidation of the dynamics of partner receptors, transducer complexes, and downstream players. Herein, we summarize current insights into the role of GPC3 in regulating cancer development through Wnt and other signaling pathways, including YAP and hedgehog cascades. We also highlight the growing body of work which underlies deciphering how GPC3 is a key player in liver oncogenesis.

## Introduction

Each year hepatocellular carcinoma (HCC) affects 750,000–1 million people worldwide and is projected to be the third most common cause of cancer death in the United States by 2030 (1). Glypican-3 (GPC3), a broadly conserved cell-surface proteoglycan, contains heparan sulfate (HS) chains connected to a core protein. Glypican regulation is linked to cell growth, differentiation and motility. GPC3 is highly expressed in >70% of HCCs but not in normal adult tissues (2). GPC3 has also been associated with poor prognosis in HCC patients (3). Taken together, this designates GPC3 as an established biomarker and indication of progression for HCC, a lethal disease for which there are limited treatment options (4, 5). While GPC3 is most notably studied in HCC, it has been implicated in other solid tumors as well (6, 7).

Wnt signaling is vital in embryonic development and tissue homeostasis (8). In adults, Wnt signaling promotes tissue renewal and regeneration. During the embryonic stage, GPC3 is widely expressed in a stage and tissue specific manner (9). GPC3 expression can be detected in the placenta and other embryonic tissues including the ovary, mammary, and lung. Several studies have shown that expression of GPC3 regulating tumor proliferation and progression through Wnt signaling cascades (10). Given that Wnt is highly hydrophobic and may require HS fragments functioning as a transporter or nano-storage unit to facilitate its activation in the extracellular microenvironment, the link between cell surface glypicans and Wnt would be highly interesting (11–14).

In this review, we highlight the role of GPC3 in cellular signaling including Wnt and other signaling pathways such as YAP (Yes associated protein), and Hedgehog (Hh) within the current scientific milieu. Due to the importance of GPC3 in multiple signaling cascades, GPC3 could have a pivotal role as a biomarker and as a potent therapeutic target in investigational immunotherapies (15). Therapeutics targeting GPC3 are in preclinical development and rigorous mechanistic insight could be pivotal in further developing a successful therapeutic strategy (15, 16).

## Biology and Structure of GPC3

Glypicans, classified among the heparan sulfate proteoglycan family, reside on the exterior cell membrane via a glycosylphosphatidylinositol (GPI) anchor and are a major part of the extracellular matrix (ECM) mediating cell-ECM and cell-cell interactions (4, 17). Glypicans comprise of a core protein attached to two HS glycosaminoglycan polysaccharide chains. The structure of glypicans is evolutionarily well-conserved and the family consists of six subtypes including GPC1-6 in mammals (18). Glypicans are typically between 60 and 70 kDa and contain a secretory signal peptide at the N-terminal and a GPI anchor at the C-terminal. All glypicans have 14 conserved cysteines, which form intramolecular disulfide bridges to connect the N terminus and C terminus, even after possible furin cleavage (19). The unique structure of glypicans provides glypicans the unique capability to store and sequester various molecules including: cytokines, morphogens, chemokines, and growth factors (15). Glypicans attract these molecules and develop concentration gradients around the ECM and cellular membrane allowing for recognition of receptors with different thresholds.

During early development, GPC3 is found in the fetal organs including: liver, lung, placenta, and kidney. In most adult tissues, GPC3 is absent or lowly expressed in most adult tissues (20). Simpson Golabi Behmel syndrome (SGBS), an X-linked overgrowth disorder characterized by a broad spectrum of clinical manifestations, is due to GPC3 loss of function mutations and primarily affects males. In SGBS patients, developmental abnormalities described include enlarged tongue, polydactyly, syndactyly, cleft palate, congenital heart defects, cystic kidneys, and vertebral fusions (21). This overgrowth phenotype has also been observed in GPC3-null mice, which expire at birth in the C57BL/6 background and share several clinical abnormalities with SGBS patients (22, 23). GPC3 is located on the X chromosome (Xq26) with Isoform 2 (GenBank Accession No.: NP_004475) being the most commonly expressed. A total of four alternatively spliced variants are documented (4, 19). The functional relevance and specificity of these isoforms is unknown. GPC3 HS chains have been shown to bind molecules including Wnt (24, 25). Interestingly, studies have suggested that the GPC3 core protein may also participate in binding Wnt as a co-receptor (26, 27). Using computational structure modeling, our group recently identified a cysteine-rich domain on the N-lobe of GPC3 for Wnt functional binding, providing evidence that GPC3 is a Wnt co-receptor that modulate Wnt/β-catenin signaling in HCC cells (28). By attracting and storing growth factors via HS chains and recognizing Wnt as a co-receptor, GPC3 acts as a cell surface glycoprotein that can modulate Wnt signaling in liver cancer.

## Modulation of Wnt Signaling via GPC3

**Figure 1:**
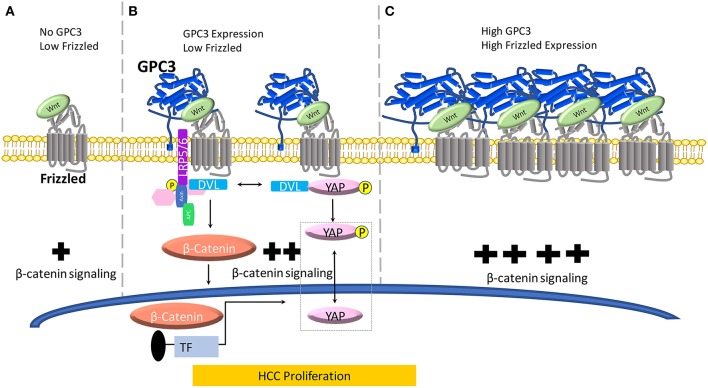
A model for the role of GPC3 in regulating Wnt in liver cancer. **(A)** When no GPC3 is present, Wnt can independently activate FZD without GPC3 co-ordination. In the absence of GPC3, there is a baseline level of Wnt/β-catenin activation in normal liver cells. **(B)** When GPC3 is upregulated in malignant liver cells (HCC), GPC3 serves as a Wnt co-receptor to attract Wnt to the cell surface via a hydrophobic groove in the N-lobe of GPC3 containing F41 and surrounding residues. **(C)** When FZD is locally concentrated and GPC3 is high in HCC cells, the Wnt/GPC3/FZD complex is formed, and Wnt signaling is amplified.

In HCC progression, activation of canonical Wnt signaling is a frequent molecular event (29). Approximately 95% of HCCs exhibit Wnt/β-catenin deregulation (30). The Wnt cascade is aberrantly activated in several human diseases including cancers and metabolic disorders. In humans, a total of 19 Wnts are secreted via autocrine and paracrine systems (31). Canonical Wnt signaling, a β-catenin-dependent process, is prompted by Wnt binding via two coreceptors: frizzled (FZD), a seven-pass transmembrane G protein coupled receptor (GPCR), and low-density lipoprotein receptor-related protein 5/6 (LRP5/6), a single-pass transmembrane receptor. There are 10 total human FZDs (32, 33). Wnt ligands bind to FZD's protruding extracellular cystine-rich domain containing the Wnt binding domain. The interaction between Wnt and FZD promotes the assembly of the FZD-LRP5/6 receptor complex (34). Conformational changes in FZD and LRP5/6, followed by phosphorylation of glycogen synthase kinase 3 and casein kinase 1 promote recruitment of Axin, an important component of the destruction complex. Consequently, DVL, a cytoplasmic protein, is recruited and binds to the C terminal tail of FZD. Thus, destruction complex, containing DVL, Axin and other binding partners, is stabilized (35–38). Axin prevents β-catenin from degradation; therefore, β-catenin accumulates in the cytoplasm, travels to the nucleus, and drives transcription of cell proliferation and survival genes (Figure 1) (39). Wnt signaling can also act in a β-catenin independent fashion, termed the non-canonical or alternative pathway (40). Since Wnt signaling is vital for many functions such as hepatobiliary functions, cell differentiation, and repair, Wnt dysregulation can result in HCC, hepatoblastoma, cholangiocarcinoma, or other liver diseases.

In HCC cell lines, overexpression of GPC3 promotes the proliferation and growth, indicating that GPC3 regulates cell surface signaling by functioning as a co-receptor for Wnt proteins (26). Interestingly, both GPC3ΔGAG (the mutant GPC3 lacking the HS side chains) and GPC3 were able to form a complex, indicating that the GPC3-Wnt complex was enabled through the core GPC3 protein. GPC3 HS chains are not required for Wnt activation, but instead, the HS chains may be important to stabilize FZD (41). These data provided initial evidence of the interaction between GPC3 and Wnt. Our laboratory reported evidence of the potential mechanism of GPC3 enhancement of Wnt3a/β-catenin signaling activity in Hep3B and other HCC cells lines by blocking GPC3 by antibodies. HS20, a human monoclonal antibody isolated using phage display technology, blocks the Wnt3a/β-catenin cascade by binding the HS chains (10). The HS20 antibody interferes with binding of GPC3 to Wnt3a and impedes access to FZD. In the same study, we showed that HS20 inhibited Wnt/β-catenin signaling in HCC cell lines and cells which endogenously express GPC3. Then, using an *in vivo* model, our group showed that HS20 has considerable antitumor activity when nude mice were inoculated with Hep3B and HepG2 cells, separately (10). In another study, the endogenous interaction between GPC3 and Wnt was confirmed in the Hep3B model (42). Additionally, the oncogenic human sulfatase SULF2, which is upregulated in over 60% of HCCs and has, 6-O-desulfatase activity in mammalian cells, can release Wnt from HS chains and form a complex with GPC3 and Wnt. This provided an indication that sulfation of HS may play an essential role for binding Wnt and other growth factors. To understand the exact mechanism for the binding motif of Wnt on the HS glycans, we and collaborators devised an array of synthetic HS oligosaccharides with differing lengths and sulfation modifications (25). We found that 2-O and 6-O sulfations were essential for Wnt binding while 3-O sulfation could enhance Wnt binding, providing direct evidence for a Wnt binding domain on the HS chains on GPC3 (25). This work also provided mechanistic insights about the size of the Wnt binding domain which we estimated to be between 6 and 8 sugar residues. Taken together, these data reasonably link GPC3, SULF2, Wnt, and FZD. However, the precise Wnt binding domain on HS is yet to be shown by structural and functional studies.

Evidence of the Wnt binding domain on the GPC3 core protein has been suggested by using the HN3 single-domain antibody (27, 43). In a recent study, we and collaborators modeled Wnt/GPC3 to predict hydrophobic areas of interest (28). We identified, phenylalanine 41 (F41), a key residue within GPC3's hydrophobic groove located in the N-lobe of GPC3. We mutated the F41 residue as F41E and found it to be critical in recognizing Wnt3a in HCC cell and mouse models (28). Furthermore, in the same study, we showed that both major parts of GPC3, the core protein and HS glycan chains, can modulate Wnt signaling (Figure 1). In a Wnt functional reporter assay, overexpression of GPC3 alone activated Wnt signaling and could be lessened by the F41E mutation, but not by eliminating HS chains (Figure 1B). Interestingly, co-transfection of GPC3 and FZD induced synergistic activation of Wnt activity. This synergistic effect was stopped by removing the HS chains of GPC3, however the F41E mutation no longer showed any effect (28). This dynamic model can conceivably connect GPC3 expression and HCC progression in which low FZD and no GPC3 represents normal liver, high GPC3 and low FZD represents early stage HCC, and high FZD and high GPC3 coordination represents late stage HCC (44) (Figure 1). When GPC3 is upregulated in malignant liver cells (HCC), possibly by chronic inflammation due to hepatitis viral infection or other etiological factors (Figure 1B) (45), GPC3 serves as a Wnt co-receptor to attract Wnt to the cell surface via the newly identified cysteine-rich hydrophobic groove in the N-lobe of GPC3 containing F41. When FZD is locally concentrated and the Wnt/GPC3/FZD complex is formed, the HS component rather than the core protein of GPC3 can serve as a bridge for the stability of the complex (Figure 1C). In this way, GPC3 may act as a bridge through its HS chains to stabilize Wnt and FZD after the Wnt/GPC3/FZD complex is formed. Thus, depending on the levels of FZD, GPC3 can promote Wnt activation through either the core protein or HS chains (Figure 1).

## GPC3, Wnt, and YAP

Early work in the *Drosophila* model implicated the Hippo signaling pathway in modulating organ size and development. In mammals, the Hippo cascade involves two main kinases Mst1/2 and Lats1/2. Once these kinases are in play, Lats1/2, phosphorylates, YAP, a transcriptional co-activator. YAP inactivation leads to downregulation of target genes including: cyclin E, diap1, and bantam. YAP has been shown to be a critical nuclear effector within Hippo signaling, however, the precise mechanism by which Hippo signaling inactivates YAP function in mammals remains unclear (46). Furthermore, recent advancements in understanding signaling pathways have indicated that the Hippo pathway suppresses liver overgrowth and HCC development. YAP function is critical in regulating cell size, tissue regeneration, and cancer morphogenesis. Studies have indicated a connection between GPC3 and Wnt via YAP however the mechanism of crosstalk between β-catenin and YAP remains undetermined (Figure 1B). Additionally, in the cytoplasm, there is evidence that YAP can regulate DVL (47). Moreover, in liver cancer, HCC tissues showed higher YAP activation, indicating a positive correlation between HCC progression and YAP activity (48).

Our laboratory used phage display technology to identify the human single domain antibody, HN3, a GPC3 target, and showed that HN3 potently inhibited HCC cell growth. When investigating the mechanism of HN3 activity, we found that phosphorylated YAP (p-YAP), the inactive version of YAP, was greater in HCC cells treated with HN3. Overall, total YAP level was reduced in HCC cells treated with HN3. GPC3 knockdown led to lower cell proliferation and reintroduction of recombinant YAP was able to rescue the cells from apoptosis triggered by GPC3 knockdown (49). The observation is consistent with our early finding that GPC3 regulated YAP signaling in HCC cells (43). When we knocked down YAP in HCC cells, cell proliferation decreased by ~50%. Upon subsequent HN3 treatment, YAP-knockdown cells did not further inhibit cell proliferation, indicating that YAP knockdown may cause acquired resistance to HN3 treatment. However, in mutant YAP overexpression cell lines where YAP is constitutively active, we reported increased cell proliferation and abatement of HN3 antagonist activity, i.e., HN3 could not inhibit cell proliferation. In a subsequent work, used a reporter assay to investigate YAP activity. In Hep3B cells, blocking Wnt via HN3-GPC3 binding also blocks YAP, overall indicating that Wnt may be involved in the upstream regulation of YAP signaling (27). Taken together, these results indicate that YAP is not only involved in HCC proliferation but also that GPC3 may act as an upstream regulator of YAP. As discussed previously, Li et al. reported that the HN3 antibody recognizes the Wnt binding site, a unique conformational epitope which is a cysteine-rich, hydrophobic groove in the N-lobe of GPC3 (28, 43). These works reasonably link YAP inactivation to GPC3 and Wnt via HN3.

## GPC3, Wnt, and Hedgehog Signaling

**Figure 2:**
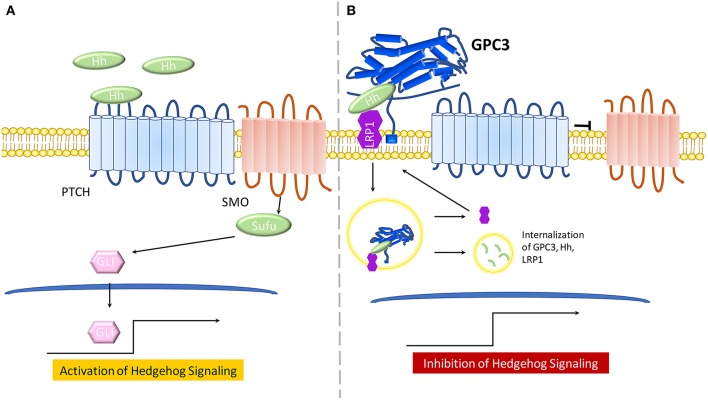
The model for the role of GPC3 in regulating Hedgehog signaling. **(A)** In the presence of Hh ligand, PTCH promotes surface localization and activation of SMO. SMO transduces the Hh signal within the cytoplasm. Protein kinases phosphorylate GLI proteins, leading to an NH_2_-terminal truncated form, which travels to the nucleus and activates Hh signaling. **(B)** GPC3 competes with PTCH for Hh binding which results in inhibition of hedgehog signaling. GPC3 binds both Shh and Ihh, resulting in the endocytosis and lysosomal degradation of the GPC3/Hh complex in the presence of LRP1.

The hedgehog (Hh) signaling pathway (Figure 2) plays a defining role in embryonic development and is highly conserved across species. Hh signaling is involved in cell growth, differentiation, tissue patterning, and vascularization. When aberrantly activated, these processes can lead to tumor growth, malignant transformation or metastasis. Thus, hyperactivation of the Hh pathway has been linked to various cancers including breast, prostate, liver, pancreatic, and brain (50, 51). In exploring the biological role of GPC3 in liver cancer (HCC) cells, GPC3 was found to promote HepG2 cell proliferation through Hh signaling (52).

The Hh signaling pathway involves recruitment of Hh ligands including: desert hedgehog (Dhh), Indian hedgehog (Ihh), and Sonic hedgehog (Shh). Any Hh ligand can initiate binding to the 12 transmembrane proteins Patched (PTCH) and various co-receptors, thus triggering Hh signaling by Smoothened(SMO) de-repression. SMO, a 7-pass transmembrane protein from the FZD family of GPCRs, mediates downstream signaling. Sufu, a cytoplasmic protein, and GLI proteins, main transcriptional effectors, cooperate to induce Hh activation and expression of Hh target genes. In the absence of Hh ligands, or in the case of Hh ligands binding to GPC3, PTCH will not be active and SMO will be repressed, thereby inhibiting Hh signaling (Figure 1B) (53, 54).

Early work in the *Drosophila* model demonstrated that glypicans are involved in regulation of Hh signaling (53). GPC3-null mice, a SGBS disease model, display increased Hh signaling activity and higher levels of Shh and Ihh (55). Further, GPC3 binds to Shh and Ihh with high affinity and competes with PTCH for Hh binding (55). Of note, the core protein of GPC3 can directly bind Hh to inhibit its signaling activity in cell culture (26). In a later study, experimental evidence demonstrated that cleavage by convertases is also crucial for GPC3 inhibition of Hh signaling (56). Low-density-lipoprotein receptor-related protein-1 (LRP1) was also shown to mediate endocytosis of the GPC3-Hh complex (57) (Figure 2B).

CD81, a cell surface tetraspanin, which facilitates Hepatitis C Virus (HCV) entry into hepatocytes, further entangles GPC3 with Hh and Hippo signaling. CD81 is main GPC3 binding partner and the GPC3/CD81 interaction modulates Hh signaling through hematopoietically expressed homeobox protein (Hhex), a transcriptional repressor (58, 59). However, 78% of HCCs do not express CD81 indicating loss of CD81 expression occurs commonly in HCCs (60). In the JM2 rat hepatoma cell line, forced expression of CD81 in the presence of high GPC3 expression, increased activation of the Hippo pathway by decreasing nuclear YAP (60). The precise connections between these signaling pathways remain unclear and future work which elucidate the interplay between YAP, Wnt, hedgehog, and other signaling players will be necessary in designing targeted therapeutics. Since the outlined signaling pathways all have underlying roles in cell growth and proliferation processes, cross regulation is an essential strategy. Therefore, key steps in Wnt, YAP and hedgehog may be connected via GPC3 and its counterparts to tightly control fundamental cellular processes as a fine-tuning mechanism (50, 54, 61, 62).

## Future Perspectives

GPC3 is clearly an important player Wnt, Hh, and YAP signaling cascades. However, fundamental questions regarding the GPC3/Wnt/FZD complex structure, intratumor heterogeneity of protein expression, and alternatively spliced variants of GPC3 in liver cancer have yet to be fully understood. Future work addressing the mechanism of GPC3 in the outlined signaling pathways would provide a more complete picture of its precise role in oncogenesis of liver cancer. Rigorous experimental interrogation of mechanism will be crucial in engineering therapies which can disrupt tumor progression. Nevertheless, as extensively summarized in other recent articles, the development of GPC3-targeted therapies has emerged with many clinical trials worldwide (15, 16, 63). These ongoing clinical trials will help define the utility of GPC3 as a target for liver cancer therapy.

## Author Contributions

All authors listed have made a substantial, direct and intellectual contribution to the work, and approved it for publication.

### Conflict of Interest Statement

The National Cancer Institute (NCI) holds patent rights to anti-GPC3 antibodies including HN3, YP7, and HS20 in many jurisdictions, including the USA (e.g., US Patent 9409994, US Patent 9206257, US Patent 9304364, US Patent 9932406, US Patent Application 62/716169, US Patent Application 62/369861), China, Japan, South Korea, Singapore, and Europe. Claims cover the antibodies themselves as well as conjugates that utilize the antibodies, such as recombinant immunotoxins (RITs), antibody–drug conjugates (ADCs), bispecific antibodies, and modified T cell receptors (TCRs)/chimeric antigen receptors (CARs) and vectors expressing these constructs. Anyone interested in licensing these antibodies can contact MH (NCI) for additional information. The remaining author declares that the research was conducted in the absence of any commercial or financial relationships that could be construed as a potential conflict of interest.

## Abbreviations

HCC: Hepatocellular Carcinoma
HS: Heparan Sulfate
YAP: Yes Associated Protein
Hh: Hedgehog
ECM: Extracellular Matrix
SGBS: Simpson Golabi Behmel syndrome
FZD: Frizzled receptor
GPCR: G Protein Coupled Receptor
LRP5/6: lipoprotein receptor-related protein
DVL: Disheveled
PTCH: Patched
SMO: Smoothened
Sufu: Suppressor of Fused.
